# Academic Performance Following Sport-Related Concussions in Children and Adolescents: A Scoping Review

**DOI:** 10.3390/ijerph17207602

**Published:** 2020-10-19

**Authors:** Mekala Neelakantan, Brinda Ryali, Maria Demma Cabral, Ann Harris, Juli McCarroll, Dilip R. Patel

**Affiliations:** 1 Department of Pediatric and Adolescent Medicine, Western Michigan University Homer Stryker M.D. School of Medicine, Kalamazoo, MI 49008, USA; Mekala.neelakantan@med.wmich.edu (M.N.); Brinda.ryali@med.wmich.edu (B.R.); mariademma.cabral@med.wmich.edu (M.D.C.); 2 Department of Library, Western Michigan University Homer Stryker M.D. School of Medicine, Kalamazoo, MI 49008, USA; ann.harris@med.wmich.edu (A.H.); juli.mccarroll@med.wmich.edu (J.M.)

**Keywords:** sport-related concussion, academic performance, quality of life, learning environment, accommodations

## Abstract

Sport-related concussions (SRC) are an increasingly common concern in young athletes, with long-term cognitive, physiological, behavioral, and psychological adverse outcomes. An estimated 1.1 million to 1.9 million SRCs occur per year in children <18 years old in the United States. The post-concussive state has demonstrated consequences in several domains, including athletics and academics, although much more research has been conducted on the former. The objective of this scoping review was to ascertain findings from published studies on the effects of SRCs on academic performance and quality of life of young student athletes. A total of 175 articles were screened within the PubMed and CINAHL databases, along with a Google search. Fourteen papers fulfilled the inclusion criteria and were analyzed in the review. Quantitative and qualitative data were collated and demonstrated the heterogeneity with which, post-concussion academic performance outcomes were measured; only 4 of the 14 studies utilized formal academic metrics such as changes in grade point average (GPA) or examination scores. While the results overall did show statistically significant implications on academic performance decline after SRC, it is clear that there remains a paucity of research determining the consequences of SRCs on academic performance in the school environment. Further research is needed to better understand how to implement accommodations in the student’s learning environment and guide return-to-learn protocols for student athletes following SRC.

## 1. Introduction

Our understanding of the mechanism, and, both short-term and long-term effects of sport-related concussion (SRC) in young athletes continue to evolve [1]. Per our literature review, SRC seems to include injuries after sports-related, athletic, or recreational activities. A generally accepted current definition of sport-related concussion is the one from the consensus statement on concussion in sports—the 5th international conference on concussion in sport [1]:

Sport-related concussion is a traumatic brain injury induced by biomechanical forces. Several common features that may be utilized in clinically defining the nature of a concussive head injury include:

- SRC may be caused either by a direct blow to the head, face, neck, or elsewhere on the body with an impulsive force transmitted to the head.
- SRC typically results in the rapid onset of short-lived impairment of neurological function that resolves spontaneously. However, in some cases, signs and symptoms evolve over a number of minutes to hours.
- SRC may result in neuropathological changes, but the acute clinical signs and symptoms largely reflect a functional disturbance rather than a structural injury and, as such, no abnormality is seen on standard structural neuroimaging studies.
- SRC results in a range of clinical signs and symptoms that may or may not involve loss of consciousness. Resolution of the clinical and cognitive features typically follows a sequential course. However, in some cases, symptoms may be prolonged.

It is important to rule out other possible causes that may result in signs and symptoms similar to concussion. These include the effects of drugs, underlying behavioral or mental disorders, or other injuries in the head, face, or neck region [1,2]. The pathophysiology of concussion involves a series of metabolic changes in the brain that result in a wide range of behavioral and neurocognitive signs and symptoms [1]. Although, most athletes show a rapid clinical recovery within 3–4 weeks following a concussion, persistent, often subtle, neurological, behavioral, and cognitive signs and symptoms are seen in many athletes. Within the context of the pediatric age group, concussions remain a common source of injury with an upward trend [3]. An estimated 1.1 million to 1.9 million sports- and recreation-related concussions are reported each year in children <18 years old in the United States [4].

Children and adolescents are at a higher risk of resulting long-term brain injury, even when the short-term symptoms of the trauma are resolved [1]. Following a concussion, metabolic changes occur at neuronal cell level; the energy stores of the already fragile brain are compromised, temporarily disrupting states of metabolism and neurotransmission that are easily worsened when exposed to a higher neurological demand too early in the recovery stage [1]. Such higher demands may come in the form of physical or cognitive exertion, such as those encountered in the learning environment in the classroom setting [1].

Return to play guidelines following SRC have predominantly focused on protocols for a gradually increasing level of physical activity. On the other hand, our understanding of cognitive rest and return to learning is less well informed by substantial research. Most current protocols provide generalized approaches, which do not necessarily take into account the needs of the individual student [5,6,7]. 

Notwithstanding our still evolving understanding of the importance of cognitive rest and implications of SRC on learning, it is generally accepted that most students following a SRC experience a varying degree of difficulty in academic work and performance [8]. Despite the abundance of observational findings of academic decline following an SRC, there is relative paucity of quantitative or qualitative research to inform return to learning guidance. Our objective was to conduct a scoping review of available literature specifically delineating the effects of SRCs on academic performance and quality of life in the school environment. 

## 2. Materials and Methods

### 2.1. Protocol

The protocol was established using the guidelines outlined by Arskey and O’Malley [9]. Further input and subsequent changes were made based on discussions within the research team, and can be provided upon request. 

### 2.2. Search

Information sources for this scoping review included the PubMed and CINAHL databases, with data limited to English-language studies published from 2010 to 2020. The most recent search was executed on 03/30/2020. The specific search terms used for the PubMed database were as follows: *((“Brain Concussion”[Mesh] OR brain concussion OR concussion OR sport-related concussion OR sports-related concussion OR mild traumatic brain injury) AND (“Adolescent”[Mesh] OR adolescent OR high school OR secondary school) AND (“Academic Performance”[Mesh] OR academic performance OR impairment OR “Educational Measurement”[Mesh]) AND (impact OR outcome OR effect)).* The specific search terms used for the CINAHL database were as follows: *Sport-related concussion AND (adolescents OR high school student OR high school athletes) AND (academic performance or academic achievement).*

### 2.3. Inclusion and Exclusion Criteria

In order to be included, articles needed to address academic performance and quality of life in the context of the post-concussion state. Data on academic performance included number of school days missed or changes in baseline academic performance (grade point average, standardized test scores, or failing grades). Case reports, reviews, comments, or letters to the editor were excluded. Articles were also excluded if participants were above the age of 19 years or past grade level 12 or if control group participants were diagnosed with an explicit underlying medical condition. 

### 2.4. Data Charting

Data extraction for the included sources of information was independently performed by two investigators (BR and MN). Papers were preliminarily ruled-in, based on their title and abstract, then formally included or excluded based on a full review of the article. A computerized spreadsheet was created by the investigators to enter data from each paper used. Extraction items included author, year of publication, country of study, type of study, purpose or objective, sample size, age range of participants, percentage of male and female participants, methods of measurement and data collection with any specific instrument models used, key outcomes, and qualitative or quantitative data categories. 

## 3. Results

### 3.1. Selection of Sources

**Figure 1:**
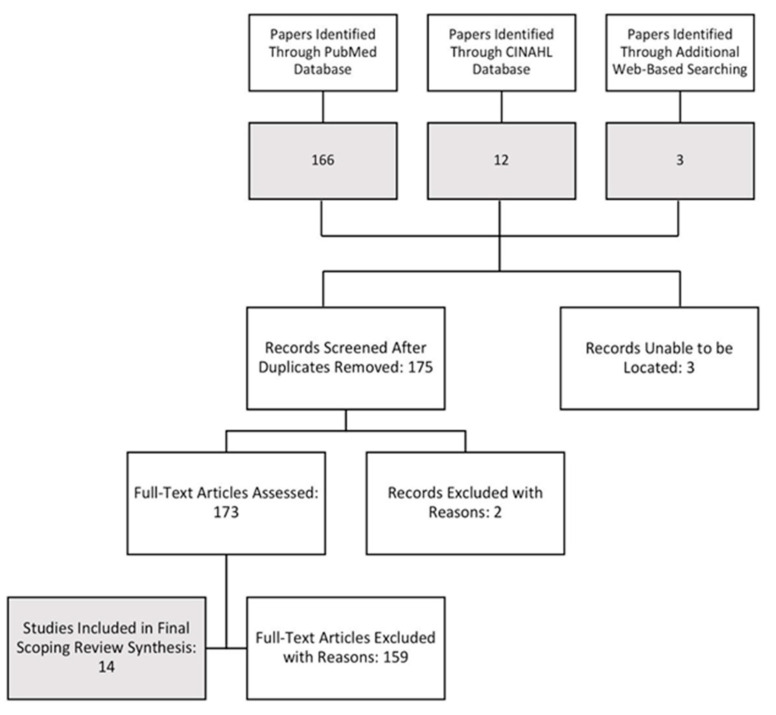
Flow diagram of records assessed for eligibility.

A total of 178 records were identified through the PubMed and CINAHL search strings, with an additional 3 studies found through other web-based sources. Three duplicates were identified between the PubMed and CINAHL databases, creating a total of 175 records that were then screened via abstract analysis. Three records were removed as the full text of articles were not available. Two records were also excluded at this level due to their irrelevance and nature of being a non-peer-reviewed website entry. The remaining 173 papers were analyzed using full-text review, with 159 being ultimately excluded for not meeting inclusion criteria, the most common being irrelevant to the study of academic performance or the upper age limit criteria. An additional 2 papers were excluded as they were not studies, rather they were webpages with general information about concussions. The remaining 14 papers were included in our final scoping review synthesis, having met all inclusion criteria without any overlapping exclusion criteria. Figure 1 depicts the flow of the study selection process.

### 3.2. Synthesis of Results.

Table 1 and Table 2 show a summary of findings of each study included in this scoping review. Table 1, containing data pertaining to post-concussion academic performance, indicates three studies that utilized qualitative survey methods, four studies that assessed academic measures (GPA, report cards, and examination scores), and three studies that utilized published inventory or achievement testing tools (Post-Concussion Symptom Inventory, Pediatric Quality of Life Inventory Version 4.0, and Wide Range Achievement Test-III) [7,10,11,12,13,14,15,16,17,18]. In regard to strength of evidence, 6 studies were classified as cross-sectional studies [6,7,11,12,18,19], 4 were cohort studies [10,15,20,21], 2 were case series [13,16], 1 was a prospective longitudinal study [14], 1 was a population-based retrospective before and after study [17].

Table 2 demonstrates studies specifically analyzing post-concussion academic accommodations along with academic dysfunction [6,19,20,21]. Outcomes for this group were presented in several ways including the quantitative numbers of school days missed, as well as types and amount of school accommodations utilized.

### 3.3. Summary of Pertinent Findings (Table 1 and Table 2)

Through a prospective cohort, Novak et al. showed that the presence of persistent post-concussion symptoms (PPCS) as compared to a lack of PPCS conferred significant decreases in total, physical, emotional, social, and school-related quality of life for at least 12 weeks post-concussion [10]. Ransom et al. used a cross-sectional survey to show that of the participants who were actively symptomatic/with neurocognitive deficits ((Rc-): 59%), 59% of students had overall concerns about school, 88% reported interference of post-concussion symptoms while in school, and 77% reported a decline in academic skills. Of participants who did not show symptoms or neurocognitive deficits ((Rc+): 16%), 16% of students reported overall concerns about school, 38% reported interference of post-concussion symptoms while in school, and 44% reported a decline in academic skills [11]. In another cross-sectional survey, Stein et al. showed that more than half of participants noted their worst symptom as being a loss of activities. The surveys demonstrated that within the “Missing Academic Activities” response theme, 7 mentioned difficulty with schoolwork and examinations, 4 mentioned the inability to go to school, and 3 mentioned feeling behind as compared to other classmates. In yet another cross-sectional study, Lowry et al. reported significantly lower self-reported GPAs after sustaining concussions than without concussions (2.95 vs. 3.14). Surveys demonstrated a decrease in self-reporting of “A” grades after sustaining concussions than without concussions (30.7% vs. 41.1%). In addition, 15.1% of participants noted at least 1 sports-related concussion [7]. Valovich et al. used a case series to demonstrate lack of consistency in academic accommodations and available assistance. They also showed that school difficulties worsened emotional symptoms post-concussion and prolonged current symptoms. More than half of the participants noted decreases in grades, difficulty in class and examinations, and overall frustration with schoolwork. 11 out of the 12 cases reported psychosocial effects of concussions in school [13]. Using a prospective longitudinal study, Alexander et al. showed that while baseline performance of rugby players with or without concussion were initially higher than controls, overtime rugby players group showed decline or plateau in academic performance [14]. In a 2016 paper, Russel et al. used a population-based retrospective controlled before-after study to show that there was no statistically significant difference in change of GPA over time between concussion and control group. They reported that concussion patients had a lower GPA at both pre- and post-concussion time periods [17]. In a 2017 paper, Russel et al. showed similar findings through a case series and reported no significant differences in GPA between pre-concussion and after concussion recovery. They also reported the median number of school days missed as 4 (IQR 2-8) [16]. In a 2019 paper, Russel et al. conducted a prospective cohort study to once again show that no significant difference existed between pre-injury vs. post-injury overall or core grades amongst SRC students. In relation to accommodations, 53.1% of students with SRC reported their school was very accommodating [15]. Following a cross-sectional study, Moore et al. reported that the arithmetic score was significantly lower in the concussion group when compared to the control group. However, no significant differences were found in total composite score, reading score, or spelling score between concussed participants and control participants [18].

Prasad et al. used a cross-sectional survey to demonstrate that students with severe TBI received high rates of academic accommodations. On the other hand, students with complicated-mild/moderate TBI showed more variability in the provision of support services [19] After conducting a single-center, cross-sectional retrospective analysis, Lopez et al. reported that out of 308 student athletes seen at a concussion clinic, 72 received school accommodations. They also determined that there were no significant differences in age, injury year, loss of consciousness (LOC), days between injury and clinic visit, or baseline ImPACT scores between students who received accommodations versus those who did not [6]. Wasserman et al. conducted a prospective study and showed that participants with concussions showed a longer lapse between returning to school than participants with extremity injuries (mean 5.4 days +/− 5.1 vs. mean 2.8 days +/− 2.6). 1 week after the injury, 42% of concussed participants received academic accommodations while 25% of extremity injury participants received academic accommodations. Based on survey results, 61% of concussed participants self-reported academic dysfunction 1 month after their injuries [20]. In another prospective study, Chrisman et al. reported that 90% of participants returned to the school environment by 9 days with the mean days until return reported as 6.7 days [21].

## 4. Discussion

Our review found that there remains a paucity of available literature that looked at the direct consequences of sports-related concussions on student academic performance and quality of life. Not only is there paucity in literature, there is wide heterogeneity in types of studies, lack of standardization in determining academic decline following SRC, and differences in age ranges. Recovery following an SRC manifests in many different ways, each requiring varying amounts of time [22,23]. Studies show that resolution of clinical manifestations of SRC occurs within 3 weeks in most athletes; however, nearly 20% of adolescent athletes continue to have symptoms for a longer period of time [24]. One paper showed that adolescent athletes who continued to play following an SRC experienced a longer time to resolution of clinical signs and symptoms [24]. It then stands to reason that the continuation of academic and cognitive demands may similarly prolong the recovery as well. Similar to our understanding of the importance of rest from physical exertion following SRC, it is equally important to understand the importance of cognitive rest and gradual return to learning [6].

Overall, the experience of adolescent athletes following an SRC in the school environment is one replete with stressors—be it directly from the academic work or from being more isolated from other students [12]. In one study analyzing the perspectives of the adolescent athlete, found that not being able to keep up with school-work, missing academic activities, and losing the ability to successfully complete activities, was distressful [7]. Several studies demonstrated an increased likelihood of a decline in measured academic performance following SRC [14,15]. Prior to resumption of academic work, it is important to assess a student’s readiness to return to learning [7]. Return to learning prior to resolution of neurocognitive deficits may contribute to further adverse consequences for the student; these include, isolation from peers, increased psychological stress, further decline in academic function, and prolongation of time to cognitive recovery [7].

Six studies used numerical measures to assess academic decline following SRC, while other studies used subjective methods such as surveys assessing adolescent or parent perspectives on academic challenges experienced while returning to school post-concussion [10,11,14,15,16,17,18]. Of the studies that reported survey-based data on adolescent or parent perspectives, all reported a significant level of concern with respect to academic difficulty [7,12,13,19,20,21]. While this data is not quantitative, it highlights the need for further research, as self-reported measures of executive dysfunction have shown to be strong predictors of academic dysfunction [22]. Further research on predictors of academic dysfunction can also help clinicians better tailor return to school learning plans and specific accommodations to the learning environment. Of the six studies that reported numerical data on academic function following a concussion, two studies reported no statistically significant differences in academic function in the post-concussion period [17,18]. However, this does not preclude the possibility of short-term decline in academic performance or long-term adverse psychological consequences [17,20]. The differences in the effects of concussions on academic function could be variably attributed to differences in accommodations provided, the time period over which academic performance was assessed, or simply a lack of sufficient data [15]. For example, Russel et al. reported no significant change between pre-concussion and post-concussion overall or core grades [15]. Appropriate accommodations in the learning environment were reported by 53.1% of participants in this study; it is likely that these accommodations played a role in preventing a significant decline in academic function [15].

In asking post-concussion participants to identify “*What is the worst thing for you about having a concussion*,” Stein et al. emphasized the severity of the challenges faced by young athletes following an SRC; free response answers included “depression issues, low energy, not being able to keep up in school” and “falling behind in school” [7]. Not only does the limited quantitative data show a significant decline in academic performance, but the Stein et al. survey indicates adverse emotional impact as a result of academic decline [7]. This concept of potential emotional impact as a result of decline in academic performance following a SRC, is supported by Novak et al. study, which reported that children with prolonged post-concussion symptoms had significantly lower scores on school quality of life measures [10].

Academic accommodations play a significant role in the rate and extent of academic recovery following an SRC [6]. Yet, academic accommodation guidelines are sparse and too generalized. While academic accommodations vary, based on the severity of difficulties in learning and the expectations of the student and parents, the accommodations are most often not individualized to meet the specific needs of the student within the context of his or her learning environment [19]. Due to the gradual decline in academic performance over time and the lack of consistent ongoing monitoring of academic progress following a concussion, many students do not receive academic accommodations when academic decline is recognized [6]. This finding highlights the need to gather more long-term objective data on the impact of SRC on academic performance. Students have found a variety of accommodations to be beneficial including reduced attendance, periodic breaks from class, avoiding noisy environments, and rescheduling or being excused from tests [17]. However, responses varied between students, highlighting the need for an individualized approach to determining academic accommodations [17].

Given that there is significant heterogeneity in the duration and severity of symptoms, mechanism of injury, and consequences of SRCs, a prudent approach is to tailor any return-to-learning protocol to individual student’s needs. Such an approach requires coordination between multiple stakeholders within the student’s learning environment—parents, athletic directors, teachers, guidance counselors, local and state-level health authorities, and student’s physician—in order to formulate the most appropriate return to learning protocol for the student [10].

While it may be a natural assumption to associate neurocognitive deficits with a decline in academic performance or learning difficulty, performance on tests that measure neurocognitive function may not be an appropriate measure to predict academic dysfunction [22]. In determining factors that may predict academic dysfunction following a concussion, Ransom et al. found that cognitive performance did not predict perceived academic decline [11].

## 5. Brief Comment on Current Practice

Following an SRC, it is crucial to have an understanding of how to safely and effectively return the student athletes to their school environment. SRCs have varying long-term and short-term effects on children and adolescents and variable course through recovery, which has direct implications on management. The pathway to recovery and return to learning after an SRC is best understood within the context of the interplay of two concepts: cognitive exertion and cognitive rest [1,24,25,26]. According to the CDC, cognitive exertion describes the imbalance of brain energy distribution in the aftermath of an SRC [26]. Instead of allowing the brain time to recover, undue exertion through a swift return to learning and cognitive activities may prevent the brain from utilizing energy to repair itself. This is where cognitive rest comes in, namely the concept of taking a step back from rigorous cognitive activity in order to provide the brain with dedicated recovery time. It is important in these instances to understand that students may recover at different rates; the care team should be consistently monitoring the student’s symptoms and tolerance level during this process.

Individualized plans for a return to the learning environment are necessary, especially with symptom severity and recovery rate varying between students suffering from an SRC). In the United States educational system, educational accommodations are a large part of this planning process, with a wide variety of options from more informal and temporary services to more persistent plans and protocols. Parents, pediatricians, teachers, academic psychologists, and social workers should work in tandem to create a plan that fits best for the patient.

Guidelines currently recommend a 4-step graduated return to school. Students first resume typical daily activities such as light reading and social interactions with family that does not trigger worsening of symptoms [1]. These activities should initially be restricted to 5–15 min with a gradual increase as tolerated [1]. Screen time should be limited during this time [1]. Once students can comfortably tolerate 30–45 min of typical daily activities, they can then begin school activities outside the classroom [1,25]. After building a tolerance to increased cognitive exertion, they can progress in a stepwise fashion to partial school days and then, finally to full school days [1,25]. Checkpoints at each step ensure that athletes are not sent back to the classroom before they are ready. If students continue to have symptoms with cognitive exertion in school, accommodations such as increased breaks between assignments, shorter assignments, providing a quiet room to finish assignments, and avoiding noisy areas such as cafeterias should be considered [1,25,26].

In the formulation and implementation of an individualized educational plan, certain symptoms of SRC may be of specific relevance in their likelihood for impeding learning. Some of these symptoms include persistent headache, inability to have good sleep, dizziness, increased sensitivity to light and noise in the immediate environment, difficulty in concentrating, and impaired short-term recall. Additionally, some specific factors may be of relevance in guiding the educational accommodations. Based on the guidelines from the US Centers for Disease Control and Prevention following are examples of such considerations [26]:

- Do some classes, subjects, or tasks appear to pose greater difficulty than others do? (compared to pre-concussion performance)
- For each class, is there a specific period after which the student begins to appear unfocused or fatigued? (e.g., headaches worsen after 20 min)
- Is the student’s ability to concentrate, read, or work at normal speed related to the time of day? (e.g., the student has increasing difficulty concentrating as the day progresses)
- Are there specific things in the school or classroom environment that seem to distract the student?
- Are any behavioral problems linked to a specific event, setting (bright lights in the cafeteria or loud noises in the hallway), task, or other activity?

## 6. Conclusions

Few studies have specifically looked at the impact on student academic performance following an SRC and even fewer have looked at the impact on quality of life measures following an SRC in the school environment. While we have a much more clear understanding of how best to return athletes back to sports following an SRC, our understanding of how best to return student athletes back to learning is limited. Most return to learn guidelines make general recommendations that do not necessarily reflect the impact of multiple factors on the course of recovery following SRC for an individual athlete. While additional research will help in our understanding of the impact of SRC on academic performance, the findings from a limited number of studies support the importance of cognitive rest, accommodations to the learning environment, and a gradual return to learning for students following SRC.

## Figures and Tables

**Table 1:** Review of studies analyzing post-concussion academic performance and quality of life with methods utilized [7,10,11,12,13,14,15,16,17,18].

Article	Author	Year of Publication	Country of Study	Type of Study	Patient Demographic	Article Purpose	Methods
**Association of persistent post-concussion symptoms with pediatric quality of life**	Novak et al.	2016	Canada	Prospective multicenter cohort	**Sample Size:** 2006 **Age or Grade Range:** Median of 11.8 years **Gender Distribution:** 1241 Male/765 Female	“To determine the association between HRQoL (health related quality of life) and PPCS (persistent post-concussion symptoms) at 4 weeks after concussion and assess the degree of impairment of HRQoL in the subsequent 12 weeks.”	PCSI Ped-sQL-4.0
**Academic effects of concussion in children and adolescents**	Ransom et al.	2015	USA	Cross-sectional survey	**Sample Size:** 349 **Age or Grade Range:** 5–18 Years **Gender Distribution:** 67% Male/33% Female	“to study the nature and extent of the adverse academic effects faced by students recovering from concussion.”	PCSI CLASS
**Young athletes’ concerns about sport-related concussion: the patient’s perspective**	Stein et al.	2016	USA	Cross-sectional survey	**Sample Size:** 121 **Age or Grade Range:** Mean of 14.8 years +/− 2.16 **Gender Distribution:** 67 Male/54 Female	“Few studies have examined the experience and concerns of the concussed athlete. The purpose of this study was to identify the most pressing concerns of athletes with concussion.”	Survey-based
**Concussion and academic impairment among U.S. high school students**	Lowry et al.	2019	USA	Cross-sectional study	**Sample Size:** 14,765 **Age or Grade Range:** Grades 9–12 **Gender Distribution:** 49.3% Male/50.7% Female	“To examine the associations between self-reported sports- and physical activity-related concussions and symptoms of cognitive impairment (difficulty concentrating, remembering, or making decisions) and self-reported academic grades”	Survey-based
**Lived experiences of adolescent athletes following sport-related concussion**	Valovich et al.	2017	USA	Case series	**Sample Size:**12 **Age or Grade Range:** 15.7 +/− 1.7 Years **Gender Distribution:** 8 Males/4 Females	“To explore the psychosocial aspects of concussion among adolescent athletes.”	Survey-based
**Mild traumatic brain injuries in early adolescent rugby players: long-term neurocognitive and academic outcomes**	Alexander et al.	2015	South Africa	Prospective longitudinal study	**Sample Size:** 96 **Age or Grade Range:** 12–18 years **Gender Distribution:** 100% Male	“To prospectively investigate differences between young adolescent male rugby players and non-contact sports controls on neurocognitive test performance over 3 years and academic achievement over 6 years.”	WISC-III final grades
**Academic outcomes following adolescent sport-related concussion or fracture injury: A prospective cohort study**	Russel et al.	2019	Canada	Prospective cohort	**Sample Size:**124 **Age or Grade Range:** Grades 9–12; Ages 13–17 **Gender Distribution:** 58.9% Male	“To compare the effects of adolescent sport-related concussion (SRC) and sport-related extremity fracture (SRF) on academic outcomes including change in school grades and school attendance; and to determine which specific academic accommodations were most helpful during recovery from these injuries.”	Report cards
**Academic outcomes and accommodations following adolescent sport-related concussion: A pilot study**	Russel et al.	2017	Canada	Case series	**Sample Size:** 33 **Age or Grade Range:** 14.3 Years +/− 1.0 **Gender Distribution:** 57.6% Male	“To examine academic achievement, absenteeism and school accommo-dations following adolescent sport-related concussion (SRC)”	Report cards PCSS
**Academic outcomes in high-school students after A concussion: A retrospective population-based analysis**	Russel et al.	2016	Canada	Population-based retrospective controlled before-after study	**Sample Size:** Concussed: 1417; Non-Concussed: 3867 **Age or Grade Range:** Ages 14–18; Grades 9–12	“To determine if academic performance was lower in the academic calendar year that students sustain a concussion compared to the previous year when they did not sustain a concussion.”	Final Grades
**A targeted neuropsychological examination of children with a history of sport-related concussion**	Moore et al.	2019	USA	Cross-sectional Study	**Sample Size:** 16 Post-concussion participants;16 control participants **Age or Grade Range:** Mean of 9.0 +/− 0.6 years for Post-Concussion Group; Mean of 9.0 +/− 0.7 for Control Group **Gender Distribution:** 10 Male/5 Female	“The current study assessed whether a target battery of neuropsychological measures of higher cognition and academic achievement would detect lingering deficits in children 2 years after injury.”	WRAT-III

**Table 2:** Review of studies analyzing post-concussion academic dysfunction [6,19,20,21].

Article	Author	Year of Publication	Country of Study	Type of Study	Patient Demographics	Article Purpose	Methods
**Long-term school outcomes of children and adolescents with traumatic brain injury**	Prasad et al.	2017	USA	Cross-sectional survey	**Sample Size:** 99 participants with TBI **Age or Grade Range:** Cohort #1: 2 months to 6 years; Cohort #2: 8 years to 15 years	“To better understand the impact of age at injury, severity of injury, and time since injury on long-term school outcomes of children with traumatic brain injury (TBI)”	Survey-based
**Academic accommodations for a countywide concussion high school program**	Lopez et al.	2017	USA	Single-center, cross-sectional retrospective analysis	**Sample Size:** 308—72 With Accommodations; 236 Without Accommodations **Age or Grade Range:** Mean of 16 years **Gender Distribution:** 47 Male/25 Female With Accommodations; 196 Male/40 Female	“To describe a symptom-based distribution of Return to Learn school academic accommodations for adolescent student-athletes recovering from sports-related concussions that can be facilitated as part of their post-injury clinical care. The aim was also to explore demographic and recovery differences between those patients who received and did not receive accommodations.”	ImPACTScoring survey-based
**Academic dysfunction after a concussion among US high school and college students**	Wasserman et al.	2016	USA	Prospective cohort	**Sample Size:** 70 Concussed; 108 Extremity Injuries **Age or Grade Range:** Median of 15.5—Concussed; Median of—Extremity Injury **Gender Distribution:** 45 Male – Concussed; 93 Male—Extremity Injury	“To determine whether concussed students experience greater academic dysfunction than students who sustain other injuries.”	Survey-based
**Concussion incidence, duration, and return to school and sport in 5- to 14-year-old American football athletes**	Chrisman et al.	2019	USA	Prospective cohort	**Sample Size:** 863 **Age or Grade Range:** 5–14 Years **Gender Distribution:** 854 Male	“To collect prospective data on concussion incidence, risk factors, duration of symptoms, and return to school and sport in 5- to 14-year-old American football participants.”	Survey-based
